# Comparative pathogenesis of peste des petits ruminants virus strains of difference virulence

**DOI:** 10.1186/s13567-022-01073-6

**Published:** 2022-07-08

**Authors:** Roger-Junior Eloiflin, Llorenç Grau-Roma, Sylvie Python, Kemal Mehinagic, Aurélie Godel, Geneviève Libeau, Artur Summerfield, Arnaud Bataille, Obdulio García-Nicolás

**Affiliations:** 1 CIRAD, UMR ASTRE, 34398 Montpellier, France; 2 ASTRE, University of Montpellier, CIRAD, INRA, Montpellier, France; 3 Institute of Animal Pathology, Department of Infectious Diseases and Pathobiology, Vetsuisse Faculty, University of Bern, Länggassstrasse 122, 3012 Bern, Switzerland; 4 Institute of Virology and Immunology, Mittelhäusern, Switzerland; 5 Department of Infectious Diseases and Pathobiology, Vetsuisse Faculty, University of Bern, Bern, Switzerland; 6 Graduate School for Cellular and Biomedical Sciences, University of Bern, Bern 3001, Switzerland

**Keywords:** Peste des petits ruminants, virulence, pathogenesis

## Abstract

Peste des petits ruminants (PPR) is an acute disease of small ruminants caused by a morbillivirus. Clinical observation of the disease in the field revealed that several species of small ruminants are affected to varying degrees. This difference in disease-related effects could depend either on the host or on the virulence of the virus strain. A previous study highlighted the difference in virulence between two strains of PPRV used to infect Saanen goats. For this breed, PPRV Morocco 2008 strain (MA08) was highly virulent while PPRV Côte d’Ivoire 1989 (IC89) strain induced mild disease. Experimental studies generally based on healthy and young animals do not permit exploration of the natural variability of the host susceptibility to PPRV. Therefore, building on the previous study on Saanen goats, the current study focussed on this breed of goat and used commercially available animals with an unknown history of infection with other pathogens. Results confirmed the previous disease pattern for PPRV IC89 and MA08 strains. Viral RNA detection, macroscopic and histological lesions were stronger for the highly virulent MA08 strain. We show here for the first time that viral RNA can be detected in the tissues of vaccinated animals. Viral RNA was also detected for the first time in serum samples, which is in agreement with the role of circulating immune cells in transporting the virus into host target organs. Thus, this study provides insight into the pathogenesis of strains of different virulence of PPRV and will help to better understand the onset of the disease.

**Supplementary Information:**

The online version contains supplementary material available at 10.1186/s13567-022-01073-6.

## Introduction

Peste des petits ruminants (PPR) is one of the most widespread and devastating infectious diseases of domestic and wild small ruminants. The huge impact on small ruminant production has led the FAO and OIE to develop and endorse the PPR Global Control and Eradication Strategy (PPR GCES), aiming to eradicate the disease by 2030 [1, 2]. Since the first recorded description of a PPR virus (PPRV)-related disease made by Gargadennec and Lalanne in Côte d'Ivoire in 1942 [3], the disease has steadily progressed over time across Africa, the Middle East, Asia and Europe [4].

PPRV is a morbillivirus that generally causes an acute disease in small ruminants characterised by high fever, oral lesions, pneumonia and diarrhoea leading to severe dehydration and often death. In the subacute form, the lesions are less pronounced and clinical signs are often limited to ocular and nasal discharge. Subclinical forms of infection can be observed in endemic areas, especially in sheep [5–8]. This means a wide range of clinical outcomes is observed, ranging from mild to acute respiratory disease and death. PPRV is transmitted mainly by direct contact. Large quantities of virus are found in nasal and ocular discharges, saliva and faeces of infected animals. Virus shedding goes on for a long period, starting from the beginning of the incubation period to the end of the diarrhoea phase. Detection of PPRV during this period can be made from swabs of ocular and nasal discharges or, less reliably, from unclotted blood samples or faeces [9–11]. PPRV detection is routinely performed with molecular biological methods such as RT-PCR or RT-qPCR, virus isolation and competitive ELISA [12]. It has been demonstrated that animals vaccinated with the known conventional vaccines do not shed the vaccine virus [13].

Clinical observation of the disease in the field has revealed that several species of small ruminants are affected to varying degrees. Goats are generally more severely affected than sheep [14]. Some wild species and other endangered wild artiodactyl populations are clinically affected by PPRV in a similar manner to domestic sheep and goats, while others show no apparent sign of infection but produce antibodies against PPRV [15, 16]. For these animals the impact of the disease remains largely unknown [17]. In recent years, the list of domestic and wild species susceptible to PPRV with or without clinical signs has been increasing [18]. Host susceptibility to infections by the same virus vary considerably depending on the host species, breeds, their immune status and certain environmental factors [19]. It is likely that, in the host-virus interaction, this difference in disease severity greatly depends on the susceptibility of the host and on the virulence of the virus [20]. Also, passages in cell cultures can affect the degree of pathogenicity of an isolate [21]. Upon infection, PPRV can be found in both lymphoid tissue (spleen, tonsil, mesenteric, sub-maxillary, bronchial, retropharyngeal and pulmonary lymph nodes) and non-lymphoid tissue (nostrils, trachea, abomasum, jejunum, ileum, cecum, lung, intestine, liver, kidney and heart) [22, 23].

A previous study highlighted the difference in virulence between two strains of PPRV when used to infect Saanen goats either by the intravenous or intranasal route [9]. For this breed, the PPRV Morocco 2008 strain (MA08) was highly virulent while the PPRV Côte d’Ivoire 1989 (IC89) strain induced mild disease. This study has therefore provided a reliable model for evaluating the susceptibility of Saanen goats to these two different PPRV strains. Alongside this study, others have described the pathogenesis of PPRV related to virulent strains. These reports have highlighted signs of the disease in animals and how to assess them, thereby providing evidence to improve our understanding of the disease [24–29]. A sequential study designed to define the early disease establishment and pathogenesis of a highly virulent PPRV strain revealed that the nasal cavity, trachea, bronchi, tongue and lymph nodes draining these tissues are positive for PPRV RNA from 1 day post-infection. After viremia and secondary replication in generalised lymphoid tissues, PPRV infects and replicates in epithelial cells [30]. However, only a few studies have described signs associated with a difference in virulence between strains [21].

Importantly, most experimental studies are based on healthy young animals, which makes difficult to explore the natural variability of host susceptibility to a pathogen [25, 26, 30]. Studies of viral and host interactions are critical for elucidating why PPRV manifests differently among species and breeds, and providing information for the design of adapted control strategies. For these reasons, we investigated in detail the clinical outcome of infection by three different PPRV strains (high virulence, low virulence and vaccine) on adult Saanen goats with unknown infection histories for other pathogens. Several key parameters such as clinical scores, lymphocyte counts, PPRV RNA presence in blood circulation and secretions, seroconversion and the appearance of neutralising antibodies and pathology were also followed during the course of the infection of Saanen goats.

## Materials and methods

### Animal experiment

Twenty-four Saanen goats ranging from 2 to 12 years old were used for this experiment. The animals were randomly divided into 4 groups (mock-inoculated, vaccinated, IC89-infected and MA08-infected) of 6 animals each with an average age of 1.98 (± 0.2), 2.58 (± 2), 3.84 (± 4.36) and 2.63 (± 1.3) years respectively. Before viral inoculation, all animals were kept in the stables for one week to allow adaption to the new environment.

Two PPRV strains differing in virulence in Saanen goats were selected for this study. The low-virulence IC89 strain was isolated by cell culture using three passages in sheep skin explant cells followed by five passages on VERO cells. The highly virulent strain MA08 was isolated by cell culture using two passages on VERO SLAM-dog cells, followed by four passages on VERO cells and one passage on VERO SLAM-dog cells. Virus solutions were first titrated on Vero cells using the Spearman–Kärber method [31, 32]. Goats were inoculated with IC89 or MA08 strains at day 0 (D0) with a total of 4 log_10_ TCID_50_ per animal by the intranasal route (IN, 1 mL/nostril) using a specific intranasal mucosal atomization device (LMA^®^ MAD NASAL™). Another group of goats was vaccinated at D0 using a subcutaneous injection with a low viral load (2.2 log_10_ TCID_50_/dose of 1 mL) of the live attenuated Nigeria 75/1 vaccine. All doses chosen to inoculate the animals were selected to be consistent with the previous study done on Saanen goats [9]. Finally, a group of animals (mock-inoculated group) was intranasally inoculated with Modified Eagle’s Medium (MEM, Sigma Aldrich, Missouri, USA) supplemented with an equivalent amount of VERO cell culture supernatant.

The goats were clinically evaluated daily during the 3 days before inoculation (D0). From D0 onwards, the clinical evaluation was done once or more times per day depending on the health condition of the animals until the end of the study (D14). Follow-up of clinical signs was carried out by the same veterinarian. For all animals, the clinical signs recorded have been summarised in Additional file 1. Animals reaching a critical clinical status were euthanised for ethical reasons. Criteria for discontinuation of the experiment included a cumulative score of 14 (or more), a score of 3 for at least one of the parameters 1, 3 or 5 (single criterion) or if signs of severe or chronic pain were observed. These signs were rolling, frequently looking or kicking at abdomen, falling over, walking backward, rapid shallow respiration, teeth grinding, grunting, vocalisation on handling, rigidity and unwillingness to move. Daily blood samples and ocular swabs (Sarstedt, Nümbrecht Germany) were collected from animals in each group until D14. Serum was extracted from blood samples collected in S-Monovette neutral tubes (Sarstedt) and stored at −70 °C until further analysis. Swabs were immersed in 750 µL of lysis buffer RA-1 (Macherey–Nagel) complemented with β-Mercaptoethanol (ThermoFisher Scientific, Massachusetts, USA) and also kept at −70 °C.

### High throughput genome sequencing of PPR virus

The virulent strains IC89 and MA08 used in this study were sequenced prior to the start of this study using a previously published protocol [33]. cDNA was synthesised from viral RNA and amplified by PCR using five overlapping fragments: F1 (4050 bp), F2 (3663 bp), F3 (3816 bp), F4 (3800 bp) and F5 (3335 bp). The synthesis of cDNA was performed with a reverse transcriptase (RT) (RevertAid RT kit, Thermo Scientific) and the specific primers for each fragment (F1 to F5), following the instructions provided with the kit. Libraries were prepared using the Nextera kit (Illumina, California, USA) according to the manufacturer’s instructions. Sequencing was performed on an Illumina MiSeq machine at the AGAP sequencing platform (CIRAD, Montpellier, France). Consensus sequences were obtained from the raw data using a bioinformatics pipeline and were compared to sequences available online for related viruses. Thereby the consensus sequence obtained for the MA08 strain was compared to the complete genome KC594074.1 of a PPRV Morocco 2008 isolate, while the sequence obtained for IC89 was compared to the complete genome EU267273.1 of a PPRV ICV89 virus strain. Single nucleotide polymorphisms (SNPs) were determined by applying the following filter: QA/AO > 10 & AO/RO > 0.01 & DP > 100 & RPL > 2 & RPR > 2 & SAF > 2 & SAR > 2. In this filter, QA represents the addition of base “A” qualities, AO (A for Alternative) represents the number of reads on the alternative base, RO (R for reference) represents the number of reads on the reference base, DP represents the depth, RPR and RPL represents “balanced” to each side (left and right) of the site and SAF/SAR represents the number of reads on the forward/reverse sequences.

### Blood cell counting

Five hundred µL of whole blood collected in EDTA Monovette (Sarstedt) tubes was used to determine the absolute amount of white blood cells and lymphocytes. The blood cell count was monitored with Vetscan^®^ (Abaxis, California, USA) using the software parameters for goats.

### Antibody detection and virus neutralisation test

Serum samples were tested for the presence of antibodies against PPRV using the IDScreen^®^ PPR competition ELISA kit (IDvet, Grabels, France). This ELISA is based on an antibody that targets the PPRV nucleoprotein and is effective against all PPRV lineages [12]. There is only one serotype of PPR virus and therefore the antibody detection methods work for all PPRV strains [34]. Virus neutralisation was assessed for positive sera as follows: positive sera were first treated at 56 °C for 30 min and diluted in twofold serial dilutions (from 1:10 to 1:640) in MEM supplemented with 2% penicillin–streptomycin and 1% GlutaMAX (Thermo Fisher Scientific). Next, 100 µL of each serum dilution was added to a 96-well plate in triplicate or quadruplicate. A virus stock of 10^3^ TCID_50_/mL of the Nigeria 75/1 vaccine strain was prepared in MEM medium and 100 µL (10^2^ TCID_50_) of this virus dilution was added to each well of the plate and incubated at 37 °C. After 1 hour, 50 μL of VERO cells suspension of 2 × 10^5^ cells/mL was added to each well of the plate. Results were obtained after seven days of incubation at 37 °C in 5% CO_2_ atmosphere. The observation of a cytopathic effect (CPE) indicated the absence of neutralising antibodies in the tested condition. Viral infectivity was further confirmed by an immunofluorescence assay based on the detection of N PPRV viral particles. Immunofluorescence was performed according to the protocol previously described [33]. Briefly, supernatants were removed and an ice-cold 80% acetone solution was added to each well. The cells were then incubated at −20 °C for 30 min. After the incubation time, the acetone was removed and the cells were washed three times with 1X PBS. Anti-PPRV N antibody (clone 38–4, CIRAD) [35] coupled to the TRITC fluorochrome was added at a 1:100 dilution to the cells and they were incubated at 37 °C for 30 min. After the incubation time, the cells were washed three times with 1X PBS and 100 µL of PBS was added prior to observation with a GloMax^®^ Discover Microplate Reader (Promega, Wisconsin, USA). Serum neutralisation (SN) titres were calculated as the reciprocal of the highest dilution of serum that shows complete inhibition of cytopathic effects in 50% of wells. The SN titres > 10 were considered as protective [36].

### PPRV RNA detection in sera, swabs and organs

Sera samples were processed directly at the Institute of Virology and Immunology (IVI, Bern) while swabs (stored in RA1 lysis buffer complemented with β-Mercaptoethanol, Machery Nagel) and organs (crushed and stored in TRIzol) were shipped and processed at the ASTRE unit (CIRAD, Montpellier). Total RNA was extracted from sera and swab samples using the IndiMag Pathogen Kit (Indical Bioscience, Leipzig, Germany) and a Thermo Scientific™ KingFisher™ Flex Purification System (Thermo Fisher Scientific). During necropsy, samples from oral mucosa, abomasum, colon, Peyer patches, lung, spleen, tonsils, tracheobronchial, mesenteric and pharyngeal lymph nodes were collected for the quantification of virus presence. Total RNA was extracted from goat organs using TRIzol (Invitrogen, Massachussets, USA). Briefly, organ pieces were harvested and ground in TRIzol (Life Technologies), and Chloroform:IAA (49:1, Sigma-Aldrich) was added to each tube. After a centrifugation step at 12 000 *g* and 4 °C for 15 min, the upper aqueous phase was transferred to a fresh tube containing 0.5 mL of 75% ethanol. After incubation for 10 min at room temperature, nucleic acids in 75% ethanol were loaded onto a NucleoSpin RNA column placed in a collection tube. The remaining RNA isolation steps were performed using the NucleoSpin RNA kit (Macherey Nagel) according to the manufacturer’s instructions. PPRV-specific RNA was quantified in sera, ocular swabs and organs samples by real time RT-PCR (RT-qPCR), by amplifying the partial end of the N protein gene using a one-step method with the ID Gene™ Peste des Petits Ruminants Duplex kit (IDvet).

### Pathology

Full post-mortem examinations were performed from all goats immediately after euthanasia. Samples from the following organs were collected for histological examination: palate, lip, tongue, pharynx, tonsil, nasal mucosa, lymph nodes (retropharyngeal, tracheobronchial and mesenteric), trachea, lung, heart, liver, spleen, urinary bladder, kidney, oesophagus, rumen, abomasum, duodenum, jejunum, ileum, cecum, colon, brain, bone marrow and uterus. When macroscopic lesions were observed, samples comprised the affected areas and, when possible, their border with normal tissue. These samples were placed in 10% formalin, routinely processed for histology and stained with haematoxylin and eosin (H&E). Gross and histological lesions typical of PPRV infection within alimentary and respiratory tracts as well as within lymphoid tissue were semi-quantified as described below.

Gross changes within the alimentary tract consisted of erosive-ulcerative lesions, while within the respiratory system the assessed parameters were changes suggestive of bronchointerstitial pneumonia. These lesions were graded from 0 to 3 as follows: 0: absence; 1: focal or a few multifocal; 2: moderate and multifocal; 3: multifocal to coalescing or diffuse. Cumulative macroscopic scores ranging from 0 to 21 were calculated for each goat by summing the scores of macroscopic lesions in the oral cavity (lip, palate and tongue), oesophagus, abomasum, large intestine and lung and the mean of each group was compared (see “Satistical analysis” section).

Within the lymphoid organs, lymphocytic depletion was the only consistent lesion observed and it was semi-quantitatively assessed histologically as follows: 0: normal; 1: mild; 2: moderate; 3: severe. For each goat, a cumulative index of lymphocytic depletion ranging from 0 to 18 was calculated by summing the scores of all assessed lymphoid organs. Within the alimentary tract, the histological presence (1) or absence (0) of erosive-ulcerative lesions was noted and the inflammation within the lamina propria was graded from 0 (absence) to 3 (severe), generating a lesional score from 0 to 4 by summing these values. The anatomically related tissues were grouped together by calculating their mean values (i.e.,: “oral mucosa lesional score” was the mean of the lesional scores in the tongue, lip, palate and pharynx; “forestomach lesional score” the mean of rumen, reticulum and omasum scores; “small intestine lesional score” the mean of the lesional scores in duodenum, jejunum and ileum; and “large intestine” the mean of cecum and colon lesional scores), and a cumulative histologic lesional score for the alimentary tract per goat ranging from 0 to 24 was calculated. Within the respiratory tract, the degree of inflammation within the nasal cavity and trachea as well as lesions of bronchointerstitial pneumonia were graded from 0 to 3 (absence to severe). For each goat, a cumulative histologic lesional score of inflammatory lesions within the respiratory tract ranging from 0 to 9 was calculated by summing the lesional histologic scores of the assessed tissues. The mean lesional scores for all these parameters in each studied group of goats were calculated and compared. In addition, the presence of any other relevant lesions was also assessed macroscopically and histologically. Table 1 summarises the assessment of the lesions made during the study.

**Table 1:** **Assessment of macroscopic and histological PRRV-related lesions.**

	Organ or tissue	Lesions
Macroscopy	Palate	Erosive-ulcerative lesions (0 to 3^a^)
	Lip	
	Tongue	
	Esophagus	
	Abomasum	
	Large intestine	
	Lung	Bronchointerstitial pneumonia (0 to 3)
	Accumulative scoring	Sum (0 to 21)
Histopathology	Tonsil	Lymphocytic depletion (0 to 3)
	Tracheal Lymph nodes	
	Retropharyngeal Lymph nodes	
	Peyer's Patches	
	Spleen	
	Mesenteric Lymph nodes	
	Accumulative scoring	Sum (0 to 21)
	Oral mucosa (tongue, lip, palate pharynx)	Inflammation within the lamina propria (0 to 3) + erosive-ulcerative lesions (0–1^b^)
	Esophagus	
	Forestomach (rumen, reticulum, omasum)	
	Abomasum	
	Small Intestine (duodenum, jejunum, ileum)	
	Large Intestine (cecum, colon)	
	Accumulative scoring	Sum (0 to 18)
	Nasal Cavity	Inflammation within the lamina propria (0 to 3)
	Trachea	
	Lung	Bronchopneumonia (0 to 3)
	Accumulative scoring	Sum (0 to 9)

^a^0: absence; 1: mild; 2: moderate; 3: severe.

^b^0: absence; 1: presence.

### Statistical analysis

Statistical tests were carried out with Graphpad Prism (GraphPad Software, California, USA). A mixed effects model (REML) was used with the Geisser-Greenhouse correction. In this model the measures recorded over time for each of the animals were considered as matched values. P-values between the mean of the groups (vaccinated, IC89 or MA08-infected animals) and the mean of the control group (mock-inoculated animals) were calculated using Dunnett’s multiple comparison test, while comparisons between the mean of the groups were performed using Tukey’s multiple comparison test. Individual’s variances were computed for each comparison. Asterisks on the graphs highlight statistical differences between the comparisons. *P-values < 0.05; **P-values < 0.01; ***P-values < 0.001; ****P-values < 0.0001. The correlation between ELISA and VSNT data was evaluated using the Pearson coefficient R-Squared calculation.

## Results

### Sequenced genomes contain a few mutations

**Figure 1:**
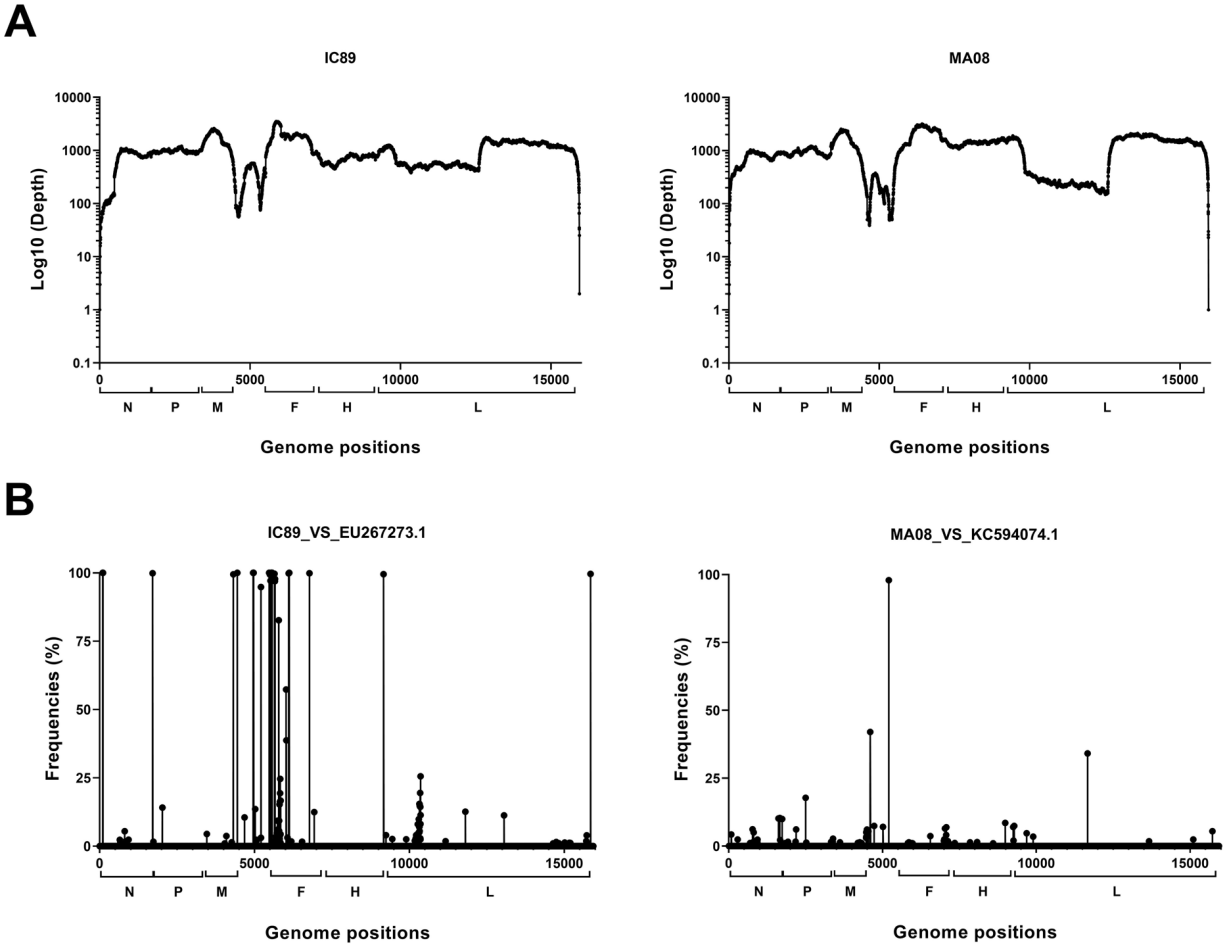
**Sequencing analysis of virulent PPRV IC89 and MA08 strains.** **A** Gene coverage (Log_10_(Depth)) obtained by illumina sequencing on each region of the viral genome. **B** Distribution of single nucleotide polymorphism (SNP) frequencies according to the genome positions. The square brackets on the x-axis represent the coding regions (CDS) of the following viral genes: N (nucleocapsid), P (phosphoprotein), M (matrix), F (fusion), H (haemagglutinin) and L (polymerase).

Sequencing of PPRV strains IC89 and MA08 was carried out to determine if there were any differences between these strains and those found in the literature. Good coverage and depth were obtained, showing that the sequencing approach chosen was effective in obtaining reads across the entire viral genome (Genbank accession numbers OL741724 and OL741725). However, the intergenic region between the M and F genes was less covered than the rest of the genomes (Figure 1A). Several high-frequency single nucleotide polymorphisms (SNPs) were found, mainly in the F gene and the intergenic region between the M and F genes, in the comparison between the IC89 and EU267273.1 genomes (Figure 1B). Twelve of the 14 SNPs found in the coding region (CDS) of the F gene caused non-synonymous amino acid mutations. SNPs in the CDS of the N gene induce a synonymous mutation while the one in the CDS of the M gene induces a non-synonymous mutation (Table 2). A single difference was found in the intergenic region between the M and F gene comparison between the MA08 and KC594074.1 genome (Figure 1B, Table 2).

**Table 2:** **Representation of high frequency SNPs**.

	Genome positions	Nucleotides EU267273.1	Nucleotides IC89	SNP Frequencies	Amino acids EU267273.1	Amino acids IC89
N CDS	1711	TTAATT	GCAGCA	99.87		
M CDS	4315	G	**A**^a^	99.46	292R	292Q
M_F intergenic region	4446	ACCGG	TTCAC	100		
	4957	ATC	AC	100		
	4963	G	C	100		
	4971	T	A	100		
	5208	AT	CT	94.84		
	5473	G	T	100		
	5499	GCGATCGCC	ACAAC	99.37		
	5509	AA	AGCACA	99.37		
	5516	T	G	99.73		
	5518	AA	ATA	97.11		
F CDS	5532	AC	**CG**^a^	100	3 T	3R
	5538	C	**G**^a^	99.65	5P	5A
	5548	A	**C**^a^	100	8 K	8 T
	5559	T	**C**^a^	99.68	12F	12L
	5563	C	**T**^a^	99.79	13S	13F
	5569	T	**A**^a^	100	15I	15 N
	5644	G	**A**^a^	99.7	40R	40 K
	5659	A	**C**^a^	96.96	45Q	45P
	5661	GC	**AG**^a^	97.75	46A	46S
	5784	C	**A**^a^	82.69	87L	87 M
	6020	A	G	57.32		
	6104	T	G	99.85		
	6122	T	C	100		
	6773	G	**T**^a^	99.89	416 W	416C

	Genome positions	Nucleotides KC594074.1	Nucleotides MA08	SNP frequencies	Amino acids KC594074.1	Amino acids MA08
M_F intergenic region	5203	TCT	CCT	95.1		

^a^Non-synonymous mutations.

### Changes in biological parameters due to PPRV infection

**Figure 2:**
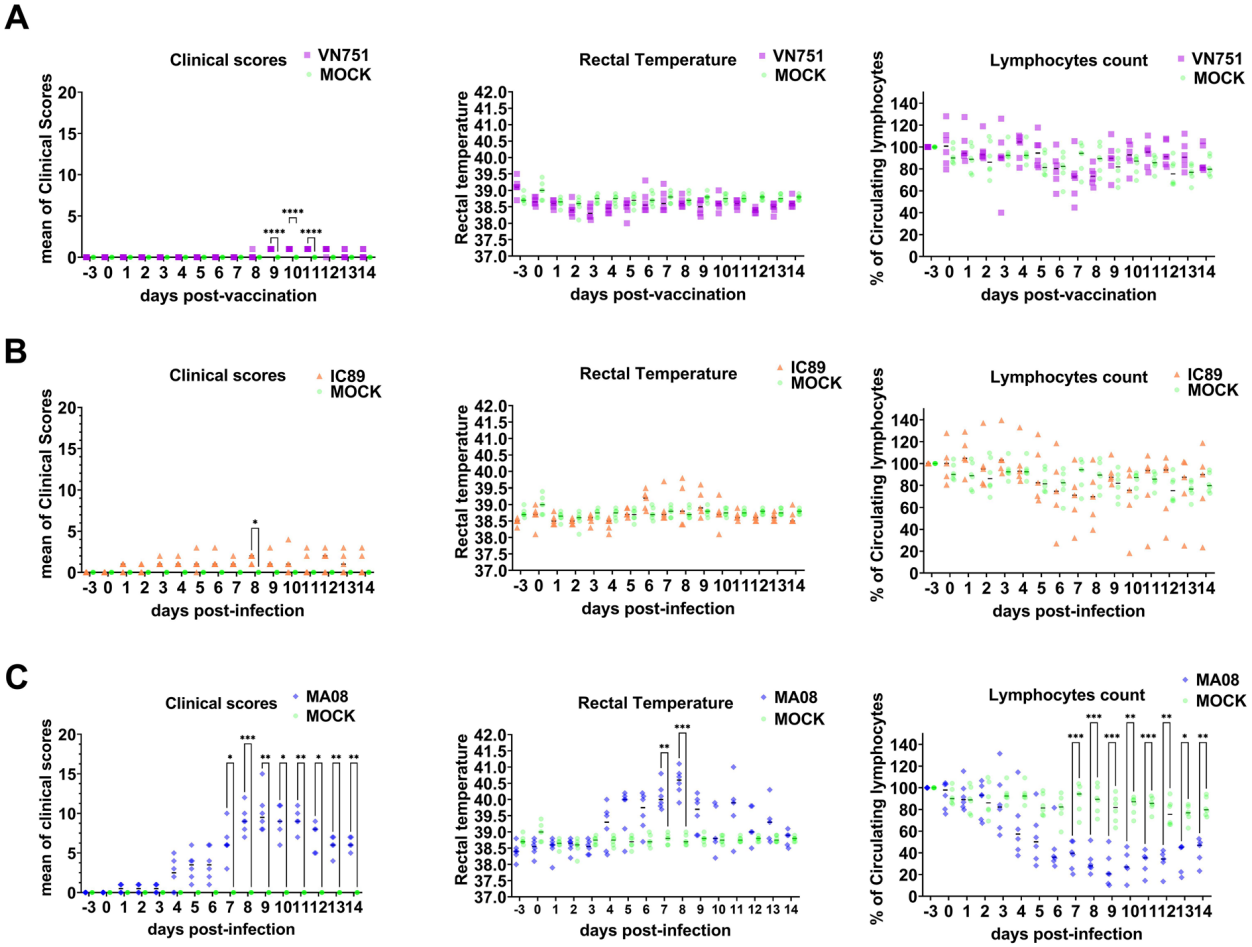
**Clinical scores, rectal temperatures and circulating lymphocyte counts.** Saanen goats were grouped in vaccinated (VN751), mock-inoculated (MOCK) or infected with PPRV strains of different virulence IC89 (mild) and MA08 (high). **A** Comparison between the vaccinated and control groups. **B** Comparison between the IC89-infected and control groups. **C** Comparison between the MA08-infected and control groups. Single dark lines in each group assessment represent the median. *P*-values were calculated using a Dunnett multiple comparison test with individual variances computed for each comparison. Asterisks highlight the statistical differences observed between the infected or vaccinated groups and the mock inoculated group. *P-values < 0.05; **P-values < 0.01; ***P-values < 0.001.

After vaccination or intranasal infection of Saanen goats, clinical signs, rectal temperature, lymphocyte count and viral load in sera were assessed. Clinical signs and rectal temperature started to increase 4 days post-infection (dpi) in animals infected with the highly virulent PPRV MA08 strain. In contrast, vaccinated, mock-inoculated and IC89-infected animals did not show any obvious signs of disease at this time. However, a slight increase in rectal temperature from 6 to 10 dpi was observed in some IC89-infected animals. A sharp decrease from 100 to 20% (*P* < 0.001) of the lymphocyte count starting at 4 dpi was observed in MA08-infected animals, while a slight and not significant decrease from 100 to 80% was observed in vaccinated, IC89-infected and mock-inoculated animals. One of the IC89-infected animals appeared to have a sharp decrease in lymphocyte count between 6 and 14 dpi (Figure 2).

### Viremia and virus shedding

**Figure 3:**
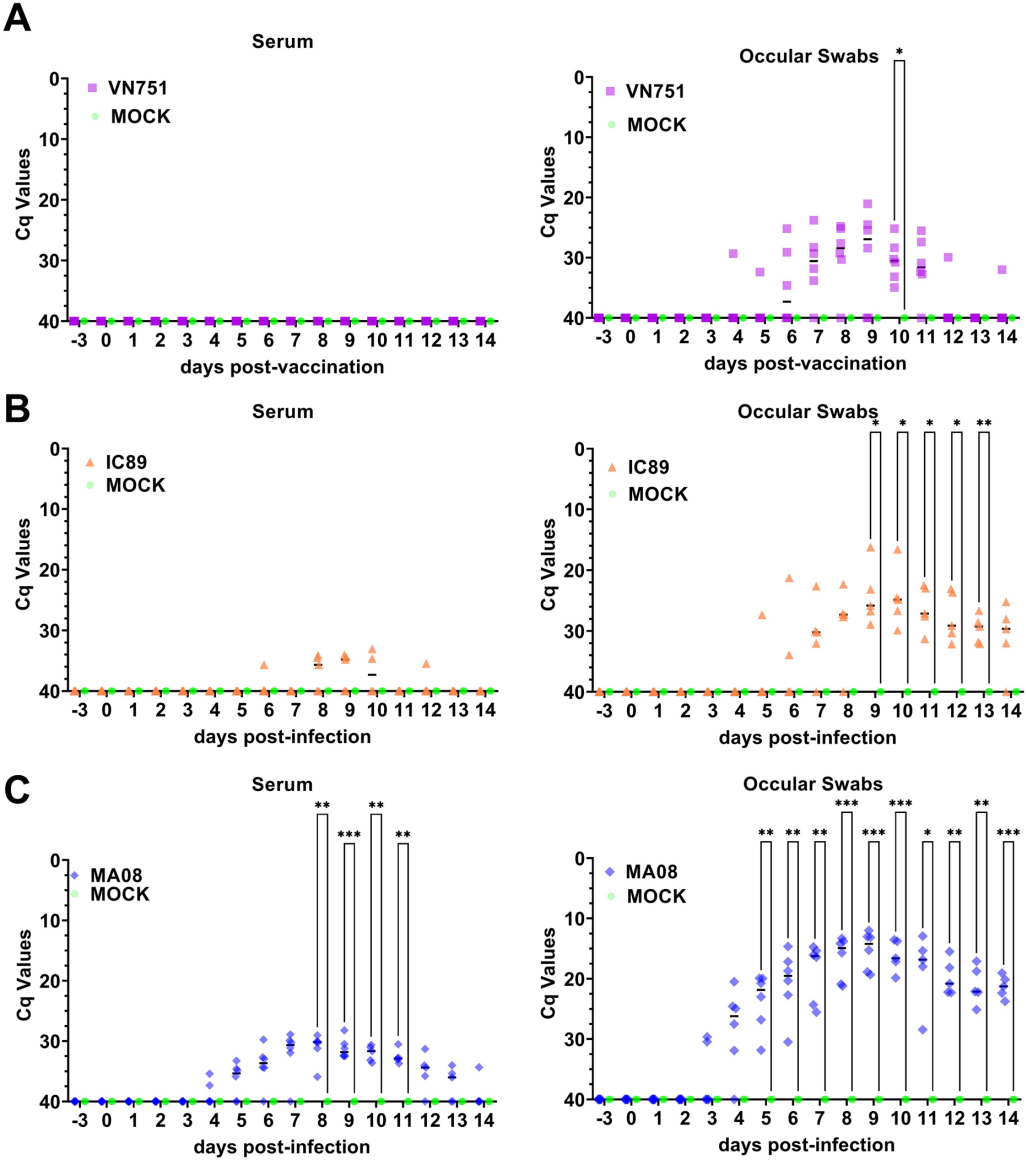
**Detection of viral RNA in serum and ocular swabs from vaccinated (A), IC89-infected (B) and MA08-infected (C) animals by RT-qPCR.** The Cq 40 value represents the absence of the viral genome in samples. Single dark lines in each group assessment represent the median. *P*-values were calculated using a Dunnett multiple comparison test with individual variances computed for each comparison. Asterisks highlight the statistical differences observed between the infected or vaccinated groups and the mock inoculated group. *P-values < 0.05; **P-values < 0.01; ***P-values < 0.001.

Serum is not a commonly used sample for the detection of PPRV RNA. We decided to include this sample because this blood fraction is used for the detection of RNA for several other viruses [37–43]. No PPRV RNA was detected in the sera of vaccinated or mock-inoculated animals, while a low and transient level of PPRV RNA was detected from 6 to 12 dpi in sera collected from some IC89-infected animals. PPRV RNA was detected in ocular secretions in some vaccinated animals from day 4 to 14, while at day 10 all samples were positive. Ocular secretions from IC89-infected animals were also positive for the presence of PPRV RNA from 6 to 14 dpi. PPRV RNA was detected on days 4 to 14 in sera collected from MA08-infected animals. Peak viremia was reached at 8 dpi and it was followed by a progressive decrease to 14 dpi. All MA08-infected animals were positive for the presence of PPRV RNA in ocular secretions from 5 to 14 dpi (Figure 3).

### Antibody responses

**Figure 4:**
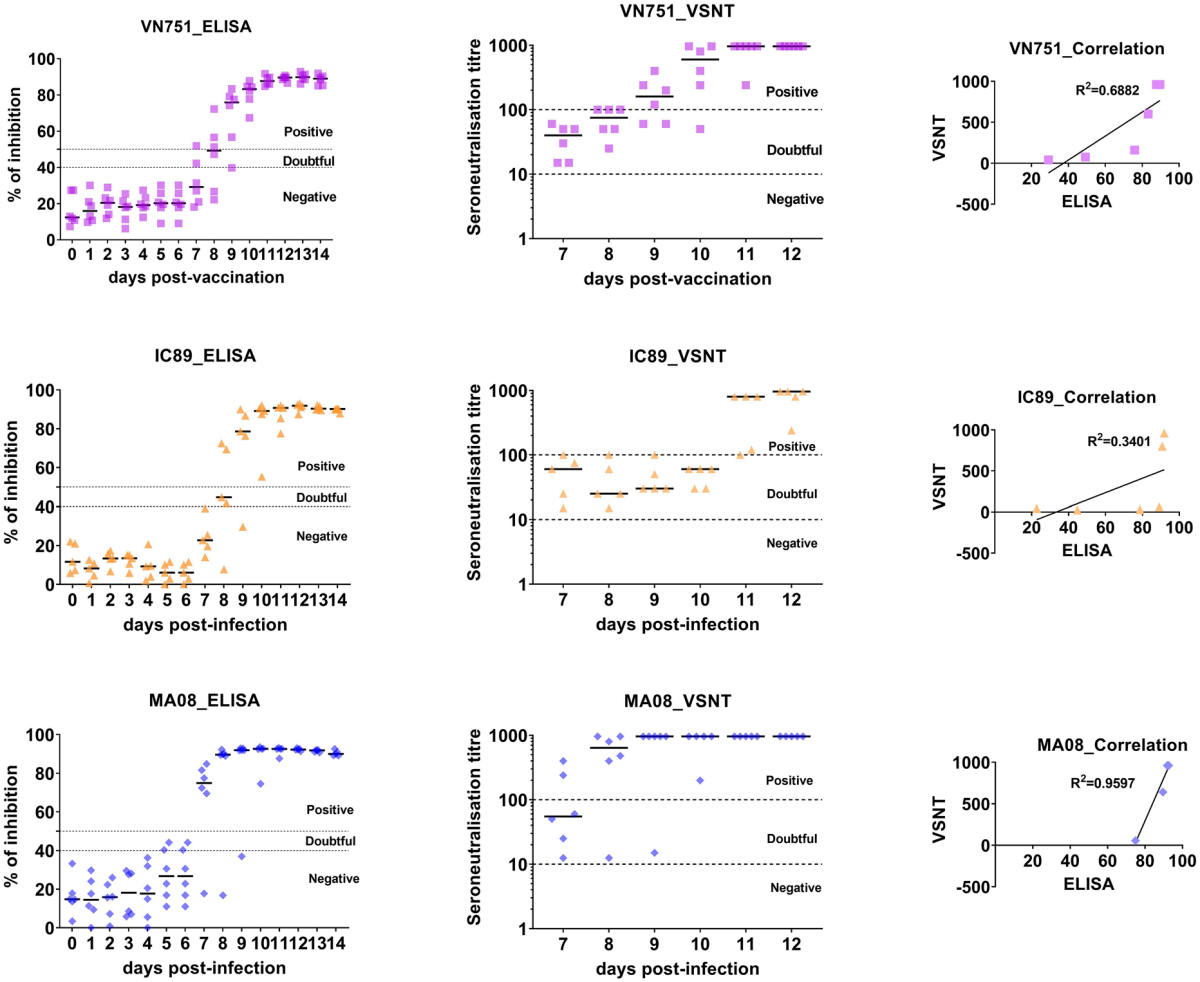
**Detection of antibodies against PPRV in (A) vaccinated (VN751), (B) IC89-infected and (C) MA08-infected animals.** Detection of PPRV antibodies was monitored by competitive ELISA (expressed in % of inhibition). Thresholds are defined as follows: from 0 to 40% = negative, from 40 to 50% = doubtful and from 50 to 100% = positive. Detection of neutralised antibodies against PPRV was monitored by VSNT. Thresholds are defined as follows: seroneutralisation titres from 1 to 10 = negative and from 10 to 1000 = positive. The correlation between ELISA and VSNT was evaluated using a Pearson coefficient R-Squared calculation.

Based on the established positivity thresholds on the virus neutralisation test, a majority of vaccinated or IC89-infected animals showed seroconversion at 9 dpi while this seroconversion occurred at 7 dpi in most of the MA08-infected animals. Neutralising antibodies were detected from 7 dpi in all vaccinated and infected animals. The SN titre peaked at 9 dpi in MA08-infected animals whereas it peaked at 11 dpi in most animals vaccinated or infected with IC89. The relationship between the SN titre and the percentage of inhibition (obtained by ELISA) were moderately positive in vaccinated or IC89-infected animals while it was strongly positive in MA08-infected animals (Figure 4).

### PPRV induced lesions are found in lymphoid and non-lymphoid organs

**Figure 5:**
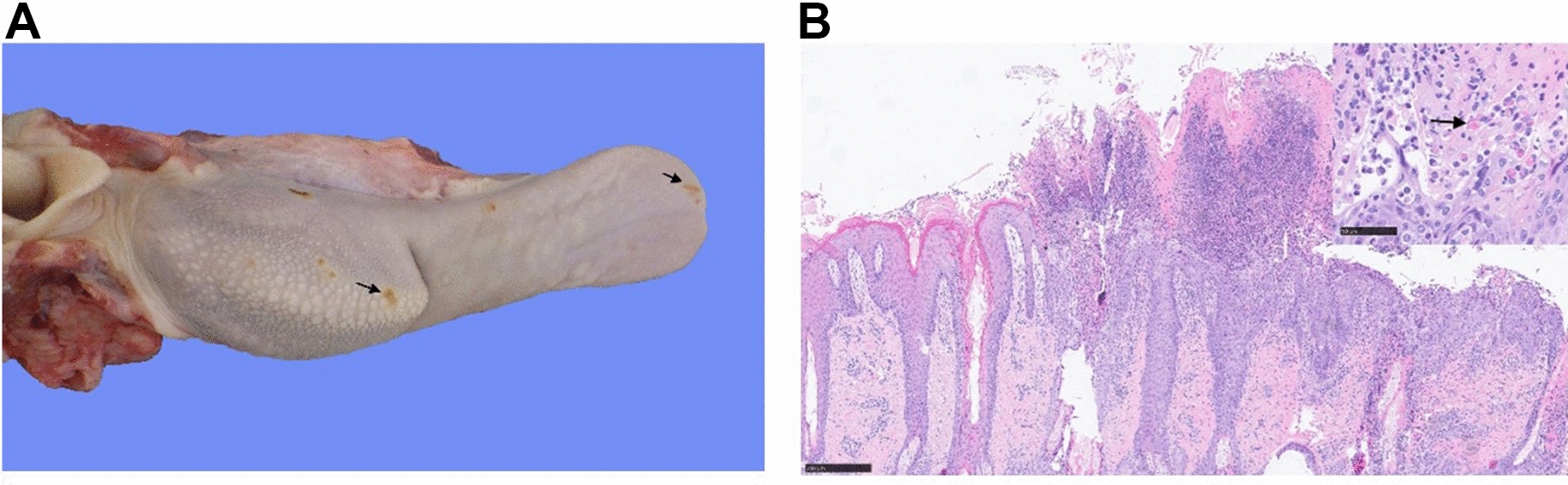
**Panel showing ulcerative lesions due to MA08 infection.** **A** Ulcerative glossitis, tongue, goat number 15, PPRV MA08-infected. There are multifocal erosions and ulcerations (arrows) in the dorsal aspect of the tongue. **B** Ulcerative stomatitis, lip, goat number 14, PPRV MA08-infected. The epithelium of the oral mucosa and the mucocutaneous junction is multifocally ulcerated and focally extensive covered and infiltrated by abundant neutrophils, lymphocytes and plasma cells. The remaining mucosal epithelium is multifocally hyperplastic. Inset: Numerous intracytoplasmic, round, eosinophilic inclusion bodies of 1 to 5 µm in diameter are present within the affected epithelium (arrow). Hematoxylin eosin stain.

**Figure 6:**
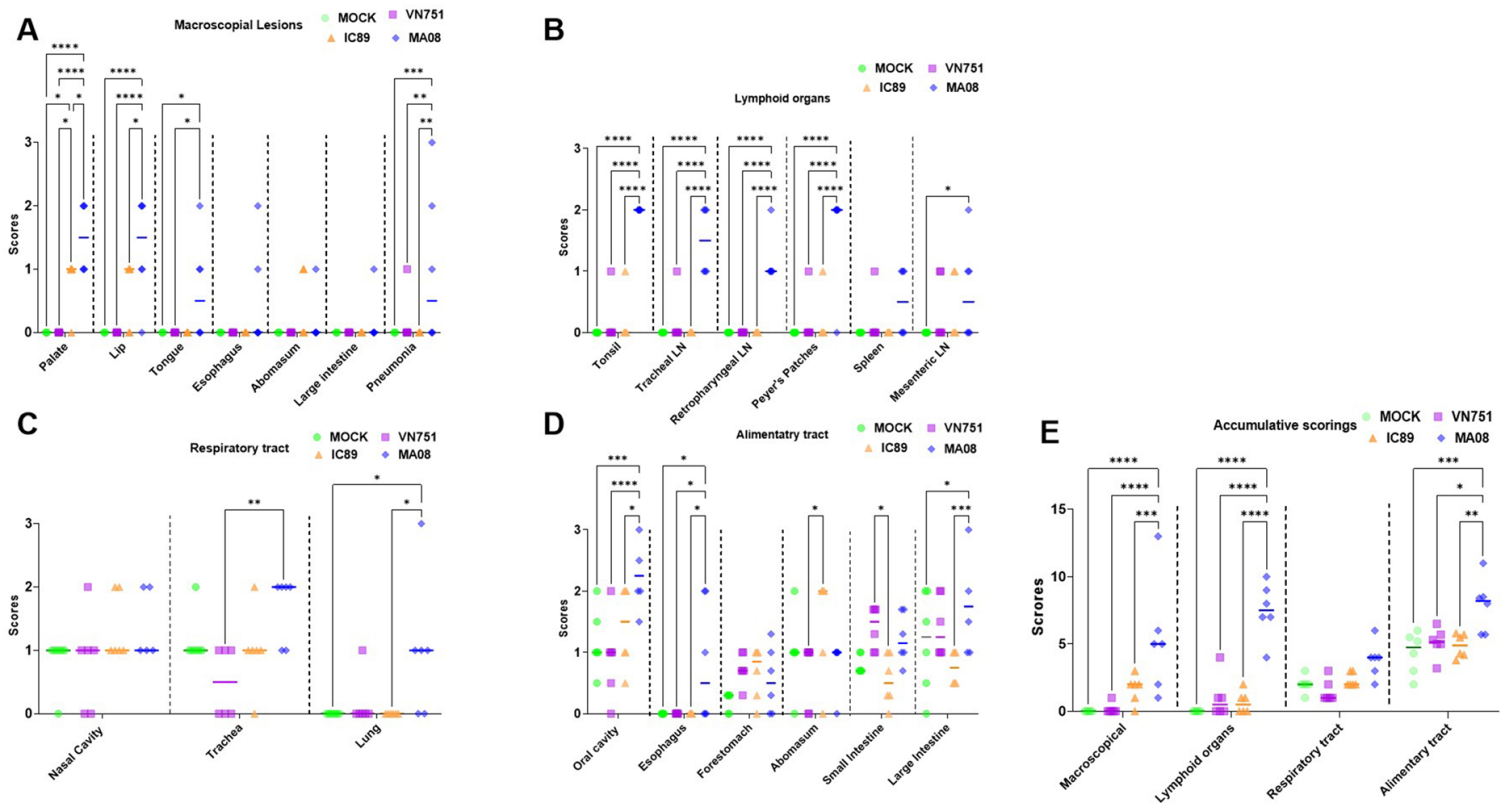
**Pathological evaluation of PPRV-induced lesions.** **A** shows macroscopic lesions in digestive and respiratory tracts, while **B**, **C** and **D** depict histological scoring in lymphoid organs, respiratory and digestive tracts. **E** represents the accumulative macroscopic and histological scoring of suspected PPRV-induced post-mortem lesions. LN: lymph nodes. Single lines in each organ or group assessment represent the median. *P*-values were calculated using Tukey multiple comparison tests with individual variances computed for each comparison. Asterisks highlight the statistical differences observed between the infected or vaccinated groups and the mock-inoculated group. *P-values < 0.05; **P-values < 0.01; ***P-values < 0.001; ****P-values < 0.0001.

Erosive-ulcerative lesions were only observed in goats infected with IC89 and MA08, and were more commonly seen within the oral cavity (all animals from the MA08 group and 5 out of 6 from the IC89 group) (Figure 5A). In the MA08 group, the lesions were of greater severity and some animals also showed lesions in the oesophagus (2 out of 6), abomasum (1 out of 6) and large intestine (1 out of 6). In the IC89 group, these lesions, when present, were always of a mild intensity and were observed only in the oral cavity or abomasum (2 out of 6 animals) (Figure 6A). Macroscopic lesions suggestive of bronchointerstitial pneumonia were seen only in animals from the MA08 group (3 out of 6). The mean of the accumulative scorings of the macroscopic lesions in the MA08 group was higher than the VN751, IC89 and mock-inoculated groups (*P* < 0.001) (Figure 6E).

**Figure 7:**
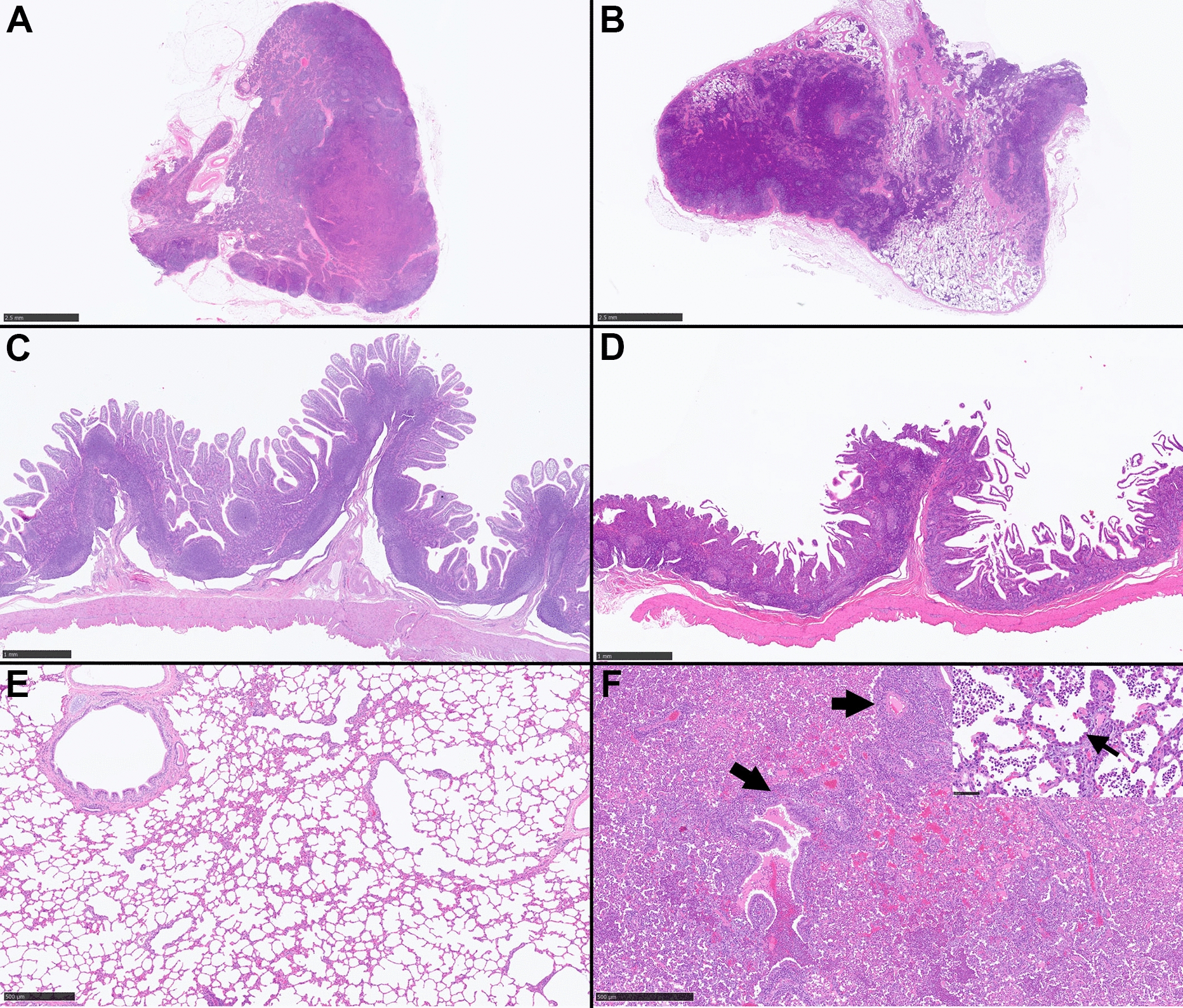
**Panel showing hematoxylin eosin stained tissues from IC89-infeced goats without histological abnormalities (left) and PPRV MA08-infected goats (right) for comparison.** **A** Retropharyngeal lymph node, goat number 9. Unremarkable. **B** Retropharyngeal lymph node, goat number 13. There is moderate depletion of lymphocytes and lymphoid follicles are multifocally poorly distinct or absent. **C** Ileum, goat number 8. Unremarkable. **D** Ileum, goat number 14. There is a moderate depletion in the Peyer’s patches. **E** Lung, goat number 9. Unremarkable. **F** Lung, goat number 13. The parenchyma is diffusely effaced by a bronchointerstitial pneumonia and a concomitant suppurative bronchopneumonia. There are abundant perivascular and peribronchiolar lymphocytes with hyperplasia of the bronchiolar epithelium (arrows) as well as neutrophils within bronchiolar and alveolar lumens. Inset: There is a prominent hyperplasia and hypertrophy of pneumocytes type 2 (arrow). Alveolar lumens contain moderate numbers of neutrophils and fewer macrophages.

Histologically, MA08-infected animals showed higher mean scores of lymphocytic depletion in most of the assessed organs as well as the accumulative scores compared to the other groups (*P* < 0.001), while no significant differences were present between vaccinated, mock-inoculated and IC89-infected groups (Figures 6B and E and 7A–D). In addition, moderate lymphocytosis was observed in the Peyer’s patches of one goat from the MA08-infected group. This lesion was, however, not observed in any other tissue or animal.

The histological lesional scoring of the respiratory tract (i.e., trachea or lung) in the MA08-infected group was significantly higher than the others (*P* < 0.05) (Figures 6C, 7E and F), although no significant differences were observed when comparing the accumulative scoring of the respiratory tract (Figure 6E). Regarding the histological lesions of the alimentary tract, the lesions found in the oral cavity (oral mucosa, pharynx and tongue), oesophagus and large intestine were discriminating between group MA08 and the other groups. The lesions found in the abomasum and small intestine showed a discrimination between the IC89 and VN751 groups. The forestomach did not show significant differences between the groups (Figure 6D). The mean of the accumulative scoring of histological lesions in the lymphoid and alimentary tract organs in the MA08 group was significantly higher than the VN751, IC89 and mock-inoculated groups (Figure 6E). In addition to the scored parameters, convincing features characteristic of PPRV infection (syncytial cell formation and/or inclusion bodies) were observed in the respiratory and/or alimentary tract of 4 goats, all of them belonging to the MA08-infected group (Figure 5B).

### Viral RNA detection in organs

**Figure 8:**
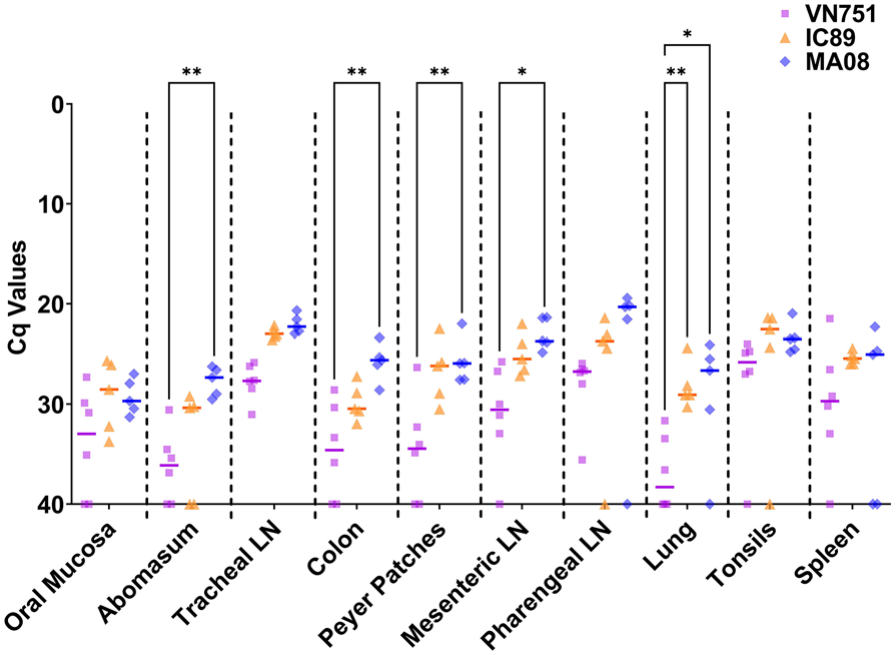
**Detection of viral RNA in lymphoid and non-lymphoid organs by RT-qPCR.** Organ samples from vaccinated (VN751), IC89-infected and MA08-infected animals were analysed for the presence of PPRV. The Cq 40 value represents absence of viral genome in samples. Single lines in each group assessment represent the median. *P*-values were calculated using Tukey multiple comparison tests with individual variances computed for each comparison. Asterisks highlight the statistical differences observed between the infected or vaccinated groups and the mock inoculated group. *P-values < 0.05; **P-values < 0.01.

Some lymphoid and non-lymphoid organs were selected according to the literature [30] and screened for the presence of PPRV RNA. Viral RNA was detected in vaccinated and IC89- and MA08-infected animals, with a higher proportion of negative samples found in the vaccinated group. Statistical differences between vaccinated and MA08-infected animals were observed in the abomasum, colon, Peyer’s patches, mesenteric lymph node and lung. In addition, a significant difference was observed in the lung samples between vaccinated and IC89-infected animals (Figure 8).

### Non-PPRV related lesions

The majority of the goats used in this study showed some pre-existing pathologies (mostly parasitic). Goats from all 4 groups had macroscopic and/or histological lesions of verminous pneumonia, trematodal cholangitis, nematodal abomasitis, intestinal nematodiasis/cestodiasis and intestinal coccidiosis (Additional file 2). In addition, one goat from the IC89-infected group suffered from an oral squamous cell carcinoma (SCC) with abscess formation and a secondary sepsis with a thromboembolic pneumonia, one goat from the MA08-infected group had a pyometra and one from the mock-inoculated group presented a mediastinal abscess. Five goats distributed among all 4 experimental groups (two being in the mock-inoculated group) showed the presence of a few multifocal lymphocytic perivascular cuffs in the brain. No relevant lesions were observed in the kidneys, heart, urinary bladder and bone marrow. Apart from the oral SCC observed in one goat, which was subsequently excluded from the study, none of the other observed lesions were severe. Considering that all the goats showed good body condition, these lesions were judged to be incidental.

## Discussion

The purpose of this study was to understand the pathological processes underlying the different clinical outcomes observed in Saanen goats infected with PPRV strains of different virulence [9]. A previous study in this breed showed that the PPRV IC89 strain induced only mild disease, whereas the PPRV MA08 strain was highly virulent and infected animals showed all the signs associated with PPRV infection. The vaccine strain Nigeria 75/1 was effective in immunising and protecting animals against PPRV infection [9, 44]. In the current study, adult Saanen goats, obtained from commercial sources, were either vaccinated with the Nigeria 75/1 vaccine or infected with PPRV strains of different virulence (IC89 and MA08). Clinical scoring during the experiment showed similar results to those observed in the previous experiment [9]. The animals in the current study infected with PPRV MA08 developed clinical signs of the disease accompanied by high rectal temperature and reached a peak of clinical signs with lymphocyte depletion, after which the animals in this group started to recover. The return to normal body temperature highlighted this recovery stage as well as the general decrease in clinical score. In recovering animals, the lymphocyte count was not restored until the end of the experiment. One animal in this group was euthanized when it reached the criteria of cessation at day 9 post-infection. Mock-inoculated, IC89-infected and vaccinated animals did not show clear clinical signs of the disease. However, a slight nasal discharge and an increase in rectal temperature were observed during the course of the experiment in some IC89-infected animals.

The slight decrease in lymphocyte count observed in mock-inoculated, IC89-infected and vaccinated animals is not necessarily linked to infection, as daily blood sampling may induce a decrease in the circulating white blood cell count [45]. Taken together, the results of this study confirm the reliability of the infection model. The PPRV MA08 strain induced acute disease as observed in previous studies [46]. The PPRV IC89 strain held in our laboratory, which induced acute disease in West African dwarf goats, caused mild to unapparent disease in Saanen goats. However, the West African dwarf breed from the rainy tropical region is more susceptible to PPRV infection than the West African long-legged goat breed from the Saharan region and this difference in susceptibility is related to the breed and not the virus [20, 47]. It has also been observed that this virus strain causes severe disease in British Saanen goats [27].

A comparison of the complete genome of the IC89 strain used in our laboratory and the genome available on GenBank reveals that the strains appear to be different, with 26 nucleotide differences separating the two genomes. One of the major issues with this analysis is that the genome of both strains was not obtained using the same sequencing method. The genome available on GenBank was obtained using Sanger sequencing while the one in our study was obtained using illumina sequencing. The un-optimised Sanger method is less effective in sequencing high GC containing sequences [48], therefore differences in the M-F intergenic region may be due to sequencing errors. The F CDS of the PPRV IC89 genome obtained in our study harbours a lot of bases in common with the F gene of other PPRV genomes, whereas the GenBank sequence presents variants in regions that are not variable in other PPRV genomes, suggesting a sequencing error in the latter (Additional file 3).

Taken together, these results indicate the possibility that most differences between sequences are due to the different method used and the Illumina sequence appears to be more reliable. It is interesting to note that one difference at position 87 in the F gene CDS seems to be specific to the sequence obtained in the present study. This mutation, which is very close to the one observed in the F protein of the measles virus, could modify the interactions of this glycoprotein and its fusogenic properties by acting, for instance, on syncytium formation [49]. New sequencing using high-throughput technology of the IC89 strain held at the Pirbright Institute (the origin of the GenBank sequence) would help identify the real nucleotide differences separating the two virus stocks. Furthermore, cell culture passages are capable of generating defective interfering particles that could make PPRV less virulent by preventing replication of the standard virus [50]. The presence of interfering particles may explain the low virulence observed in the PPRV IC89 strain at our disposal. This hypothesis should be further investigated by comparing the virulence of the two IC89 strains in a single study.

The samples collected during the current experiment were analysed for the presence of virus and antibodies. The results indicate that virus circulation in MA08-infected animals influences the dynamics and level of neutralising antibodies. Antibody levels rise earlier depending on the initial amount of circulating virus. However, total and neutralising antibodies to PPRV were detected even in the absence or presence of small amounts of circulating virus in sera collected from vaccinated or IC89-infected animals. The decrease in lymphocyte count and the high viremia and increased antibody response in MA08-infected animals may suggest that B cells are not affected by the lymphopenia or that there may be activation of antibody responses independently of T cells. The role of antibodies is crucial in controlling the disease, as clinical signs and viremia decreased within days of their onset, coinciding with the rise of neutralising antibodies.

Early presence of MA08 RNA in excreta may play a role in the probably rapid transmission of this strain, with animals excreting the virus before showing signs of the disease [51]. The low circulation of the IC89 strain in the bloodstream may be related either to the inability of this strain to infect white blood cells or to a mechanism that may occur during the initial transport of the virus to the proximal lymph nodes. No significant decrease in leukocytes was induced in the present experiment and a lower replication rate was observed when peripheral blood mononuclear cells were infected in vitro with PPRV IC89, which may support the first hypothesis [52]. The presence of IC89 viral RNA in ocular excretions, also observed in previous experiments with West African dwarf goats and Saanen goats [15, 27], points towards the maintenance of an epithelial tropism for this strain. Therefore, the interactions between epithelial cells or epithelial resident antigen-presenting cells and low virulent strains need to be studied to better understand the mechanisms behind this low virulence.

Unexpectedly, vaccine RNA was detected in ocular excretions from vaccinated animals. In the current study, viral RNA was detected in swabs from 6 to 11 days after vaccination, although the amounts of RNA were lower than those seen in animals infected with either MA08 or IC89. Other recent publications have not shown any evidence of the presence of viral RNA in ocular excretion from vaccinated animals, but less sensitive molecular methods were used [9, 53]. On the other hand, we have received confirmation from the Pirbright Institute that results similar to ours had been obtained in some of their previous experiments (Satya Parida, personal communication). The presence of RNA does not mean the presence of live virus. Unfortunately, we could not attempt to isolate live virus from the ocular swabs as the samples were inactivated to allow for their shipment. Attempts from other labs have failed (Satya Parida, personal communication). Moreover, no clinical signs or lesions were observed in vaccinated animals in this study and the detection of vaccine virus RNA occurred mostly after the appearance of antibodies at 8 dpi. These results still provide support for an inability of the vaccine to be transmitted by animal-to-animal contact, as was proven during the development of the vaccine strain [44]. Our findings again underline the need to carry out such studies in animals of a broad spread of ages and species. Researchers, notably those working in the field with vaccinated animals, should be aware of the possibility, although low, of detecting PPR genetic material in vaccinated animals during a short window of time, and be reminded that it does not put into question the safety and efficacy of the PPR vaccine.

The systematic scoring of macroscopic and histological lesions during necropsy allowed us to discriminate, to some extent, between the studied groups. Macroscopic lesions showed higher lesional scores in IC89-infected and MA08-infected animals than in vaccinated and mock-inoculated animals. These data indicate that some of the parameters assessed are useful to differentiate between the groups of goats infected with the highly virulent PPRV MA08 strain from those infected with the low-virulence PPRV IC89 strain and, in turn, to discriminate these two groups from the rest. Specifically, these parameters are macroscopic erosive-ulcerative lesions in the alimentary tract, macroscopic lesions suggestive of bronchointerstitial pneumonia, lymphocytic depletion of lymphoid organs and mucosal inflammatory lesions of the alimentary tract. The presence of virus and virus-related lesions observed in several studies have been confirmed for highly and weakly virulent strains [22, 29, 54–56]. A recent sequential study on the pathology of PPR in black Bengal goats showed that in the early stage, the virus multiplied mostly in the lymphoid organs of the pharyngeal region and caused extensive lymphoid destruction and hemorrhages. Subsequently, the virus spread to other organs causing necrotic and hemorrhagic lesions, as well as the virus localized in the upper respiratory, oral and intestinal mucosa resulting in catarrhal, erosive, and ulcerative lesions [23]. Post-mortem evaluation in the present study confirmed this pattern in animals infected with the highly virulent PPRV MA08 strain. Interestingly, the vaccine strain was also found in all the tested organs, suggesting that, although not detected in sera, the vaccine could be carried silently in the body by immune cells. As far as we know, this is the first time that viral RNA has been detected in tissue from vaccinated animals.

Finally, this study validates the infection model and may help to find discriminatory signs of the disease. To our knowledge, this is the first time that the PPRV genome has been detected in sera. However, a comparison with the detection of viral RNA in whole blood could not be undertaken, which could have made the results obtained even more significant. These new results also indicate that the treatment of suspected PPR-positive sera must be rigorous to avoid any risk of transmission of the disease. Sera collected from infected animals with rinderpest virus (RPV) have been considered to be RPV Containing Material (RVCM), although the risk of RPV presence in sera was negligible. A protocol for heat treatment established by the FAO and OIE has proven to be very effective in mitigating this risk and could be used to treat sera collected from PPRV-infected animals [57].

Neutralising antibodies are essential to control virus circulation in the bloodstream. The highly virulent MA08 strain succeeded in inducing typical clinical signs of the disease, macroscopic and histological lesions and a prolonged shedding in ocular secretions in infected animals. The low-virulent IC89 strain acted weakly, inducing only nasal discharges and a slight transient viremia in infected animals. This strain actively shed and induced macroscopic and histological lesions but in a milder way compared to the highly virulent strain. Patterns of detection of PPRV genetic material in serum supported observations of different immune responses of the host to PPRV strains of different virulence. As expected, the vaccine strain did not induce any clinical signs of the disease including or tissue lesions, however presence of viral RNA was observed for the first time in ocular excretions and organs from vaccinated animals. The high sensitivity of the method used made it possible to detect a low PPRV RNA level, for a short period, in vaccinated animals. This detection does not implies a potential risk of spreading the vaccine strain, as a large number of past experimental studies and field work has confirmed that vaccinated animals do not transmit the vaccine to contact animals [44]. Further studies are needed to better understand the mechanisms behind this phenomenon.

## Supplementary Information

**Additional file 1. Recording of clinical signs.**

**Additional file 2. Diagnosis of other pathologies (not related to PRV) in the goats studied.** The majority of goats used in this study showed pre-existing pathologies (mostly parasitic, but also in some cases of bacterial origin) which may interfere in the assessment of certain immunological parameters. Saanen goats were grouped as vaccinated (VN751), mock-inoculated (Mock) or infected with PPRV strains of different virulence, IC89 (mild) and MA08 (high). Fractions represent the number of positive animals per group of 6 goats.

**Additional file 3. PPRV F protein alignment.** Sequences A, B, C and D represent respectively the PPRV IC89 genome obtained in our study, the PPRV IC89 genome available on GenBank, the PPRV MA08 genome available on GenBank and the PPRV MA08 genome obtained in our study. The remaining sequences in the alignment are PPRV genomes obtained from GenBank. Highlighted amino acids represent some of the differences observed between sequences in Table 1.
