# Supplementary material for: Comparative pathogenesis of peste des petits ruminants virus strains of difference virulence
Source: Vet Res. 2022 Jul 8;53:57. doi: 10.1186/s13567-022-01073-6 (PMC9270740; doi:10.1186/s13567-022-01073-6)
Supplement: Supplementary file 1 — Additional file 1. Recording of clinical signs. [file 13567_2022_1073_MOESM1_ESM.docx]

| \| Parameter \| Criteria \| Score \| \| --- \| --- \| --- \| \| 1. Liveliness / Walking \| - Attentive (curious, stands up immediately) \| 0 \| \| - Slightly reduced (stands up hesitantly, but without help) \| 1 \| \| - Tired, gets up only when forced to, lies down again and/or ataxia \| 2 \| \| - Recumbent, does not stand up for more than 24 h, unable to walk \| 3* \| \| 2. Appetite \| - Greedy, hungry \| 0 \| \| - Eats slowly \| 1 \| \| - Does not eat, but sniffs food \| 2 \| \| - Does not eat at all, shows no interest in food \| 3 \| \| 3. Body temperature \| - Physiological body temperature (39-40.5 °C) \| 0 \| \| - Fever (+0.5 to +1.5 °C from baseline) \| 1 \| \| - High fever (>+1.5 °C from baseline) \| 2 \| \| - High fever for >5 days \| 3* \| \| 4. Nose and eyes \| - Normal moisture, no discharge \| 0 \| \| - Clear, slight aqueous discharge from eyes or nostrils \| 1 \| \| - Important lachrymal or nasal discharge, nasal crusts or pustules \| 2 \| \| - Large turbid or haemorrhagic nasal discharge \| 3 \| \| 5. Respiratory system \| - Frequency < 40/min, barely visible chest movement \| 0 \| \| - Frequency 40-60/min, slightly enhanced chest movements, sporadic coughing \| 1 \| \| - Frequency >60/min, slightly enhanced chest movements, coughing attacks \| 2 \| \| - Frequency >60/min or dyspnoea for >3 consecutive days \| 3* \| \| 6. Digestive system mucosa \| - Normal labial and perineal/vulva mucosa \| 0 \| \| - Light labial lesion, perineal/vulva congestion \| 1 \| \| - Moderate labial lesions, perineal/vulva purpura \| 2 \| \| - Severe labial lesions, perineal/vulva necrosis \| 3 \| \| 7. Diarrhoea \| - Normal faeces \| 0 \| \| - Soft, unformed faeces \| 1 \| \| - Watery diarrhoea \| 2 \| \| - Haemorrhagic diarrhoea \| 3 \| |
| --- | --- | --- | --- | --- | --- | --- | --- | --- | --- | --- | --- | --- | --- | --- | --- | --- | --- | --- | --- | --- | --- | --- | --- | --- | --- | --- | --- | --- | --- | --- | --- | --- | --- | --- | --- | --- | --- | --- | --- | --- | --- | --- | --- | --- | --- | --- | --- | --- | --- | --- | --- | --- | --- | --- | --- | --- | --- | --- | --- | --- | --- | --- | --- | --- | --- | --- |
