# Supplementary material for: Comparative pathogenesis of peste des petits ruminants virus strains of difference virulence
Source: Vet Res. 2022 Jul 8;53:57. doi: 10.1186/s13567-022-01073-6 (PMC9270740; doi:10.1186/s13567-022-01073-6)
Supplement: Supplementary file 2 — Additional file 2. Diagnosis of other pathologies (not related to PRV) in the goats studied. The majority of goats used in this study showed pre-existing pathologies (mostly parasitic, but also in some cases of bacterial origin) which may interfere in the assessment of certain immunological parameters. Saanen goats were grouped as vaccinated (VN751), mock-inoculated (Mock) or infected with PPRV strains of different virulence, IC89 (mild) and MA08 (high). Fractions represent the number of positive animals per group of 6 goats. [file 13567_2022_1073_MOESM2_ESM.docx]

| Groups | \| *Verminous pneumonia* \| \| --- \| | Trematodal cholangitis | Nematodal abomasitis | Intestinal nematodiasis/ cestodiasis | Intestinal coccidiosis | Lymphadenitis | Mild encephalitis |
| --- | --- | --- | --- | --- | --- | --- | --- | --- |
| VN751 | 4/6 | 2/6 | 3/6 | 1/6 | 1/6 | 2/6 | 1/6 |
| IC89 | 5/6 | 3/6 | 3/6 | 1/6 | 1/6 | 1/6 | 1/6 |
| MA08 | 5/6 | 2/6 | 4/6 | 0/6 | 3/6 | 0/6 | 1/6 |
| Mock | 6/6 | 5/6 | 6/6 | 6/6 | 4/6 | 2/6 | 2/6 |
