# Supplementary figures and images for: Comparative pathogenesis of peste des petits ruminants virus strains of difference virulence
Source: Vet Res. 2022 Jul 8;53:57. doi: 10.1186/s13567-022-01073-6 (PMC9270740; doi:10.1186/s13567-022-01073-6)

| 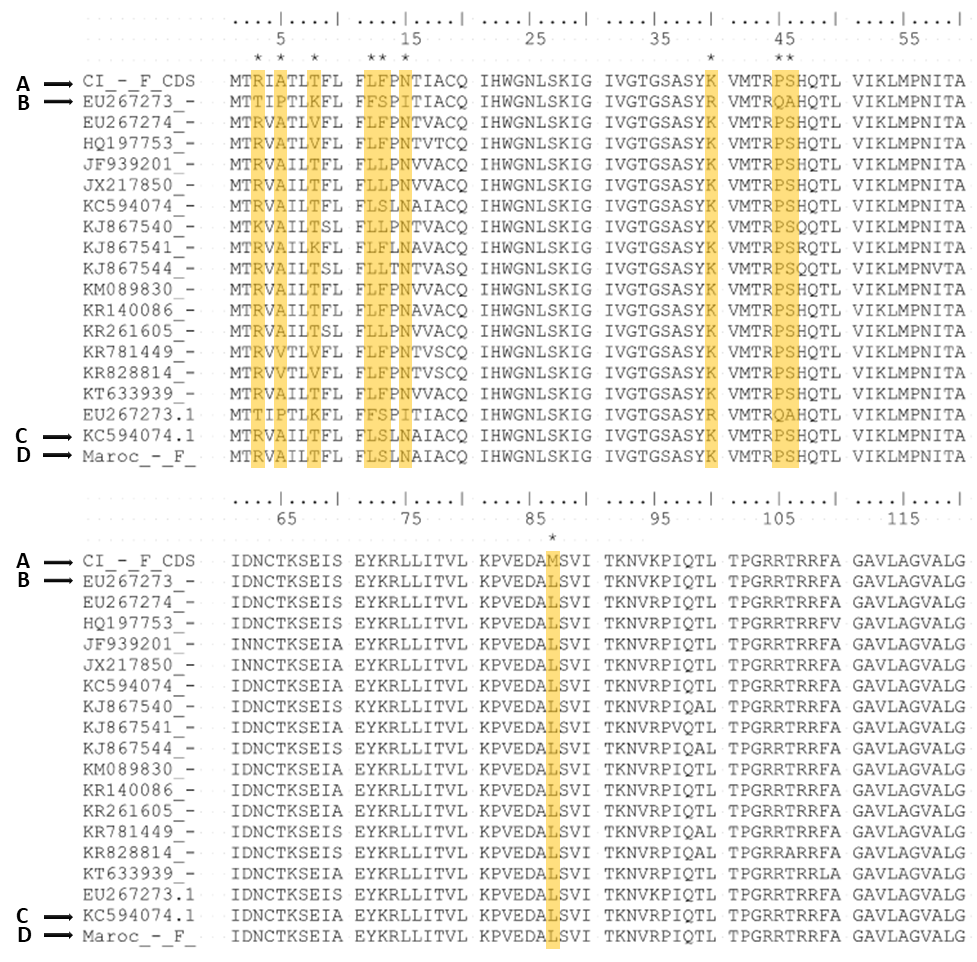 |
| --- |
|  |

Supplement: Supplementary file 3 — Additional file 3. PPRV F protein alignment. Sequences A, B, C and D represent respectively the PPRV IC89 genome obtained in our study, the PPRV IC89 genome available on GenBank, the PPRV MA08 genome available on GenBank and the PPRV MA08 genome obtained in our study. The remaining sequences in the alignment are PPRV genomes obtained from GenBank. Highlighted amino acids represent some of the differences observed between sequences in Table 1. [file 13567_2022_1073_MOESM3_ESM.docx]
